# Study of Partially Transient Organic Epidermal Sensors

**DOI:** 10.3390/ma13051112

**Published:** 2020-03-02

**Authors:** Yuanfen Chen, Reihaneh Jamshidi, Reza Montazami

**Affiliations:** 1 College of Mechanical Engineering, Center on Nanoenergy Research, Guangxi University, Nanning 530004, China; 2 Department of Mechanical Engineering, University of Hartford, West Hartford, CT 06117, USA; jamshidi@hartford.edu; 3 Department of Mechanical Engineering, Iowa State University, Ames, IA 50011, USA

**Keywords:** soft electronics, organic electronics, transient electronics, epidermal electronics, tattoo sensor, printable electronics

## Abstract

In this study, an all-organic, partially transient epidermal sensor with functional poly(3,4-ethylenedioxythiophene) polystyrene sulfonate (PEDOT:PSS) conjugated polymer printed onto a water-soluble polyethylene oxide (PEO) substrate is studied and presented. The sensor’s electronic properties were studied under static stress, dynamic load, and transient status. Electrode resistance remained approximately unchanged for up to 2% strain, and increased gradually within 6.5% strain under static stress. The electronic properties’ dependence on dynamic load showed a fast response time in the range of 0.05–3 Hz, and a reversible stretching threshold of 3% strain. A transiency study showed that the PEO substrate dissolved completely in water, while the PEDOT:PSS conjugated polymer electrode remained intact. The substrate-less, intrinsically soft PEDOT:PSS electrode formed perfect contact on human skin and stayed attached by Van der Waals force, and was demonstrated as a tattoolike epidermal sensor.

## 1. Introduction

Transient electronics are designed to operate over a predefined period of time, then self-deconstruct and vanish when transiency is triggered. These devices, especially soft transient electronics, have a wide range of potential applications in biomedical research [1,2,3,4,5] and in environmental monitoring [6,7]. In recent years, researchers have developed a variety of soft transient electronic devices ranging from electronic components [5,7,8,9,10,11] to integrated systems [12,13]. Typically, soft transient electronics are fabricated by the deposition of an active inorganic functional layer on a soft organic substrate. These inorganic functional materials, mostly metallic nano-/micromaterials, form rigid and brittle layers; therefore, the topology of the functional layer in many cases is designed so that it accommodates strain [14,15]. Such designs fall into two main categories. The first is to intentionally prestrain the substrate and/or the printed structure before regular use; thus, surface waves and buckles generated in the first cycle accommodate subsequent cycles of stretching [16,17]. The second is to laterally or topographically pattern the thin films so that global tensile strains are converted to local bending strains [18,19,20,21]. Though the topology of the inorganic functional layer is carefully designed, the mismatch of mechanical properties between the rigid inorganic layer and the soft organic substrate usually leads to sensor failure.

An approach to solve the mismatch-induced failure problem of the mechanical properties is to implement all-organic materials. The key concept and challenge are to utilize functional polymers that can accommodate strain through their molecular structure and morphology while partially maintaining their electrical properties [14]. The application of intrinsically soft materials could simplify fabrication, and enhance mechanical compliance and robustness [22,23]. In addition, the organic nature of all-polymer electronics has several advantages, including oxidefree interfaces [15] and tunability by synthesis [24,25]. Soft all-organic electronics that use intrinsically soft functional materials have received increasing attention in recent years. Liang and colleagues reported an elastomeric polymer light-emitting device using a polyphenylenevinylene derivative as the emissive [26]. Lipomi’s group investigated the effects of structural parameters of a series of poly(3-alkylthiophenes) on their mechanical properties [23]; then, they demonstrated a stretchable organic solar cell that could be conformally bonded to a hemispherical surface [22]. Bao’s group studied the electronic and morphological attributes of poly(3,4-ethylenedioxythiophene) polystyrene sulfonate (PEDOT:PSS) on stretchable poly(dimethylsiloxane) (PDMS) substrates [19]. In another study, they reported a highly stretchable and conductive polymer by adding a variety of enhancers to PEDOT:PSS [27]. Each of the all-organic electronics mentioned above were designed to last for the long term and are nontransient electronics. Combining intrinsically soft organic functional material with transient technology to achieve soft transient electronics not only enhances mechanical compliance, but also further expands potential applications of transient electronics, especially in biomedical areas [28,29]. For instance, a soft “tissuelike” transient epidermal sensor forms conformal contact with skin and provides more reliable results [30,31,32]. In particular, Inal et al. inkjet-printed PEDOT:PSS on tattoo paper, and then transferred the all-polymer electrode onto human skin as a temporary tattoo sensor that could be applied to measure electromyography [32]. However, applications of intrinsically soft functional materials in soft transient electronics remain scarce [15].

In this work, a partially transient organic epidermal sensor was studied. Combining all-organic electronic and transient technologies, an intrinsically soft functional PEDOT:PSS electrode was electrohydrodynamic-jet (E-jet)-printed onto a water-soluble polyethylene oxide (PEO) substrate. PEDOT:PSS was applied as a functional layer for its desired properties, such as high conductivity [27,33,34,35], chemical stability [33,36,37], printability [38,39,40,41], and noncytotoxicity [42,43]. A polyethylene oxide (PEO) film was used as a substrate for its hydrophilicity, biocompatibility, and flexibility attributes [43]. E-jet printing was applied as the fabrication technique because of its high-resolution [44,45], low-cost [46], and drop-on-demand-printing [41,47] characteristics. In this study, the mechanical and electrical properties, along with the transiency, of the epidermal sensor were investigated. Proof-of-concept soft transient strain/stress sensors are demonstrated and characterized. In particular, the functionality of a substrate-less electrode (with a substrate layer completely dissolved in water) as a tattoo epidermal sensor is demonstrated. The partially transient all-organic epidermal sensors presented in this study can be applied as a flexible sensor as a whole, or as a tattoo epidermal sensor with a substrate-less functional electrode.

## 2. Experiments

### 2.1. Materials

PEO (*M_w_*: 400,000 g·mol^−1^) and PEDOT:PSS (3.0–4.0% in H_2_O) were purchased from Sigma Aldrich (St. Louis, MO, USA). Dimethyl sulfoxide (DMSO) was purchased from Fisher Chemical (Lenexa, KS, USA).

### 2.2. Printing of PEDOT:PSS Conductive Patterns on a PEO Substrate

In our study, 1 g of PEO was added to 20 mL of deionized (DI) water and stirred at 50 °C for 3 h. The clear solution was then cooled to room temperature, casted onto a plastic mold, and dried at ambient conditions for 24 h. Cured film was peeled off of the mold and used as the substrate for printing conductive patterns. The thickness of the substrate was approximately 60 μm. To evaluate the printing ability of PEO substrate, contact angle measurement was done by dropping 5 μL PEDOT:PSS solution on PEO substrate, then measured the contact angle using a contact angle measuring device (SDC-200, Sindin, Shenzhen, China).

**Figure 1:**
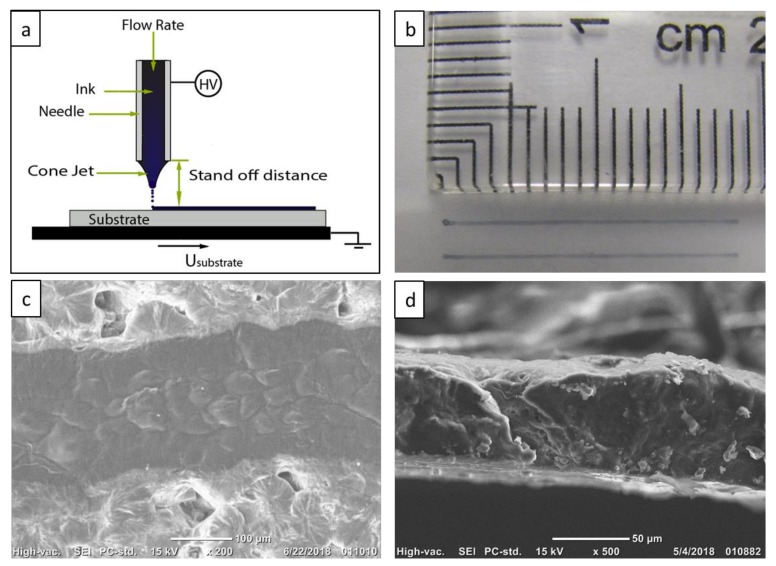
(**a**) Schematic of electrohydrodynamic jet (E-Jet) printer; (**b**) image of the all-polymer electrodes taken with a charge coupled device (CCD) camera; (**c**) electrode image taken with SEM; (**d**) image of cross-section area of electrode taken with SEM.

An in-house-made E-jet printing system (Figure 1a) was used for printing PEDOT:PSS ink into conductive patterns (electrodes) on the PEO substrate. As shown in Figure 1a, the printer consisted of two main parts, a steel nozzle and a moving stage. The nozzle had an inner diameter of 210 μm, with controllable standoff distance from the substrate. Standoff distance was maintained at 0.2 mm for all samples discussed in this report. The substrate was mounted on the x–y plane of the printer with four degrees of freedoms on the x–y plane. The velocity of the stage was kept at 2.5 mm/s during printing. A DC potential difference of 2.1 kV was applied across nozzle and substrate. To prepare the printing ink, PEDOT:PSS was diluted into 0.6–0.8 wt % in DI water. Then, 2.5% volume ratio of DMSO was added to enhance the electron conductivity of PEDOT:PSS [27,48]. The ink was then supplied into the nozzle through a 1 mL syringe loaded onto a syringe pump (Kent Scientific Corporation) through Teflon tubing. Flow rate was maintained at 2 μL/min. For each electrode, printing was repeated five times to obtain suitable thickness and conductivity.

To study the morphology of the printed patterns, a scanning electron microscope (SEM) (JCM-6000PLUS NeoScope Benchtop, JEOL, Tokyo, Japan) was used. Electrodes were freeze-fractured in liquid nitrogen to prepare samples for cross-section imaging.

### 2.3. Mechanical Characterization

The all-organic electrodes were characterized for their behavior under both static and dynamic mechanical stress using a dynamic mechanical analyzer (DMA−1, Mettler Toledo, Columbus, OH, USA). The DMA was loaded with tension clamps with a 10 mm sample opening. Static testing was performed on force-controlled mode for a range of 0–6 N at a rate of 0.2 N/min. Dynamic testing was performed under displacement-controlled mode at 3, 2, 1, 0.1, and 0.05 Hz frequencies to examine the response time, and a displacement range of 100–400 μm to test the reversible stretching threshold. All mechanical characterizations were isothermal. Clamps were insulated to prevent short-circuiting when electrical measurements were taken.

### 2.4. Electrical Characterizations

The electrical properties of printed electrodes were investigated under different conditions, including under stress, during degradation, and when used as epidermal sensors. Measurements were taken on a potentiostat (VersaSTAT 4, Princeton Applied Research, Oak Ridge, TN, USA). A bias potential of 1 V was applied during the experiments.

## 3. Results and Discussion

### 3.1. Printed All-Organic Electrodes

The contact angle between PEDOT:PSS ink and PEO substrate was measured to be 55.653 °C, as shown in Figure S1, confirming the printing ability of PEO substrate. Thus the all-organic electrodes were fabricated by printing PEDOT:PSS ink onto a PEO substrate as described in Section 2. A typical electrode array is shown in Figure 1b, with a length of 17.5 mm, a width of 180 μm, and average thickness *t* of 10 μm. Resistance *R* of each electrode was approximately 20 kΩ. Deduced from Equation (1), the conductivity of the printed PEDOT:PSS electrode was calculated to be 486 S/m. The conductivity of the PEDOT:PSS electrode could be increased by post-treatment of the film, such as by annealing the film at an elevated temperature, or by washing the residual of PSS chains with water/methanol [35,49]. The printed electrodes presented in this study were not post-treated because that PEO substrate is not physically/chemically stable in water/methanol and in high-temperature environments.

Figure 1c shows an SEM image of an all-organic electrode (top view) showing that the printed electrode consisted of three bands: a granular structure band in the center, and two transition bands on the sides. The granular structure band was thought to be the ink-jetting area, of which each grain was formed by an ink drop. One fact showing this is that a grain size of approximately 50 μm was smaller than the needle diameter, which agreed with the E-jet printing characteristic [47,50,51]. In addition, the grain shapes agreed with the mechanism that an ink drop would deform from a round shape to an elliptical shape towards the moving direction of the substrate. The transition region was anticipated to be the result of wetting/drying dynamics of the printing process and the interactions between the aqueous ink and the water-soluble PEO substrate during the printing process. Figure 1d shows that the cross-section of the printed electrode had an arc shape, which could be explained by the drying dynamics.

(1) $$\sigma =\frac{1}{\rho }=\frac{l}{R\cdot A}=\frac{l}{R\cdot w\cdot t\;}$$

### 3.2. Strain–Electric Resistance Correlations

To establish correlations between the applied stress, strain, and electrical resistance of the all-organic electrodes, electrical resistance was characterized under both static and dynamic stress.

#### 3.2.1. Static Stress

**Figure 2:**
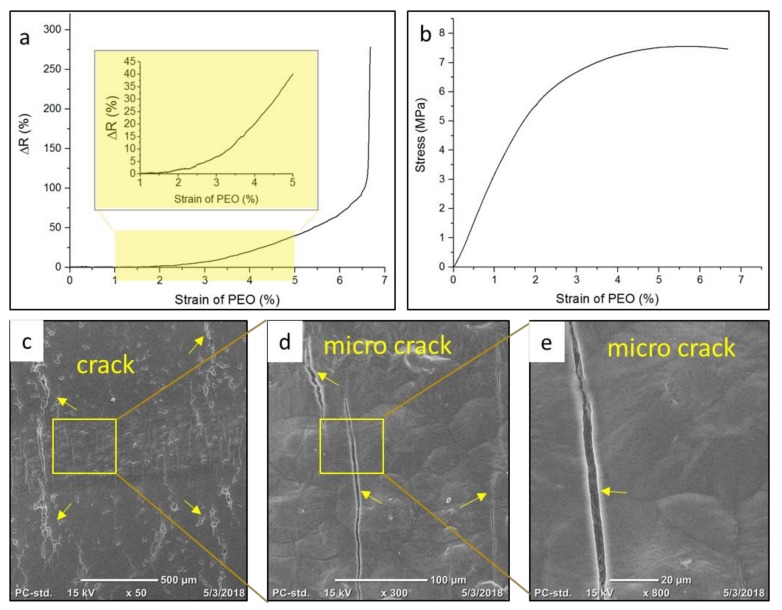
(**a**) Relative resistance changed as function of static strain; (**b**) stress–stain curve of polyethylene oxide (PEO) substrate; (**c**–**e**) SEM image of electrode after being stretched for 7%. Cracks and microcracks marked by yellow arrows.

Resistance of an electrode, similar to that presented in Figure 1b, was examined in response to strain due to a force of 0–6 N increasing at a step size of 0.2 N/min. The load condition at each step was treated as static. As presented in Figure 2a, resistance was measured and monitored as a function of PEO substrate strain. An inset graph was added to better show the increasing trend for strain below 5%. Resistance remained approximately unchanged for up to 2% strain, increased gradually within 6.5% strain, and then increased with a sharp slope thereafter. As shown in the stress–strain curve in Figure 2b, the PEO substrate remained linearly elastic within 2% strain, then turned into plastic, and yielded at about 6% strain. Figure 2c–e show images of the electrode after 7% strain. At this strain, cracks formed in the PEO substrate, and microcracks formed in the PEDOT:PSS electrode. Considering the fact that PEDOT:PSS electrodes consisted of nanoscale regions of conducting PEDOT-rich areas surrounded by insulating PSS-rich and PEDOT-rich areas, several conducting PEDOT segments entangled along a long PSS chain [52,53,54]. The electrical property of the organic electrode in response to static load is explained as follows. Within the elastic region of the substrate (*ε* < 2%), strain inside the printed electrode was accommodated by the long PSS chain rearrangement within the PEDOT-rich regions, and resistance remained almost unchanged. As strain increased further (2% < *ε* < 6%), disconnections between PEDOT-rich regions increased gradually when the PEO substrate underwent plastic deformation; thus, resistance increased gradually. When the substrate started to yield (*ε* ≈ 6.5%), cracks formed on the substrate and initiated the formation of microcracks within the PEDOT:PSS electrode, which, in turn, resulted in a dramatic increase in resistance.

#### 3.2.2. Dynamic Load

**Figure 3:**
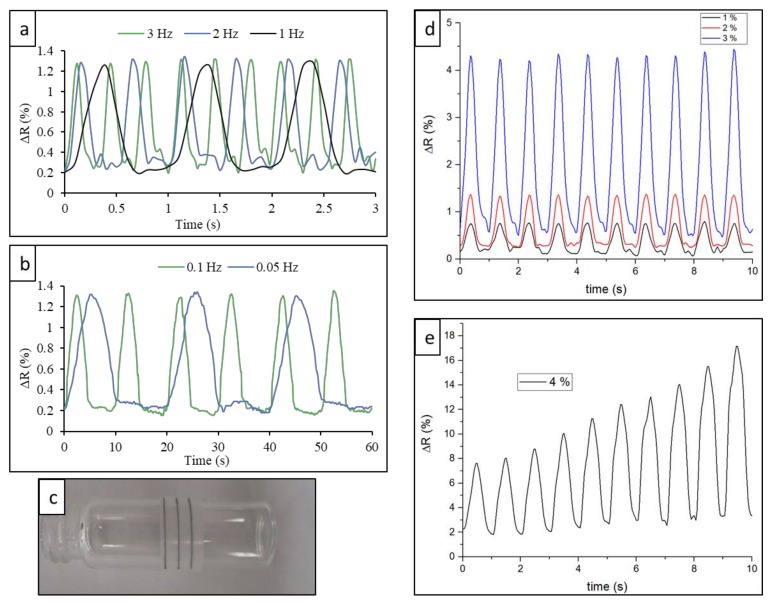
(**a**) Resistance fluctuations of a polymer electrode for 200 μm displacement at 3, 2, and 1 Hz frequencies; (**b**) resistance fluctuations of a polymer electrode for 200 μm displacement at 0.1 and 0.05 Hz frequencies; (**c**) flexible electrode bend around a glass tube of 1 cm diameter; (**d**) resistance fluctuations at 1 Hz for 100, 200, and 300 μm, respectively; (**e**) resistance fluctuations at 1 Hz for 400 μm.

Dependence of electrical resistance on dynamic load was investigated at different frequencies and strains to evaluate the time response and reversible stretching threshold of the all-organic electrodes. Frequency–resistance correlations are presented in Figure 3a,b. Stacking plots show the normalized resistance fluctuations of the electrode for 200 μm displacement (*ε* = 2%) at frequencies of 3, 2, 1, 0.1, and 0.05 Hz, respectively. The overall behavior of relative resistance fluctuations was almost identical for the five different frequencies. This suggests the frequency-independent reversibility of electrical conductivity in this frequency range. The time-response performances validated the potential applications of the presented all-organic electrode as an epidermal sensor, such as monitoring pulses (frequencies in the range of 1–3 Hz), respiration activity, and finger and arm movements (usually lower than 1 Hz). This frequency-independent reversibility deemed to fail with 400 μm displacement (*ε* = 4%). Figure 3e shows the relative resistance fluctuation at 1 Hz for *ε* = 4% where strain–resistance correlation was irreversible, with a rising baseline at each cycle. Such behavior is attributed to the plastic deformation of the PEO substrate.

Resistance fluctuations of electrodes at 1 Hz frequency for different (100, 200, and 300 μm) displacements (corresponding to *ε* = 1%, 2%, and 3%, respectively) are presented in Figure 3d. The correlation between strain and resistance, as expected, was direct. As strain increased, relative resistance fluctuations increased.

Figure 3c shows an array of electrodes wrapped around a glass vial of 10 mm in diameter to demonstrate the flexibility. The electrodes were detached and rewrapped several times, and no change in electrode performance was observed.

### 3.3. Transiency

**Figure 4:**
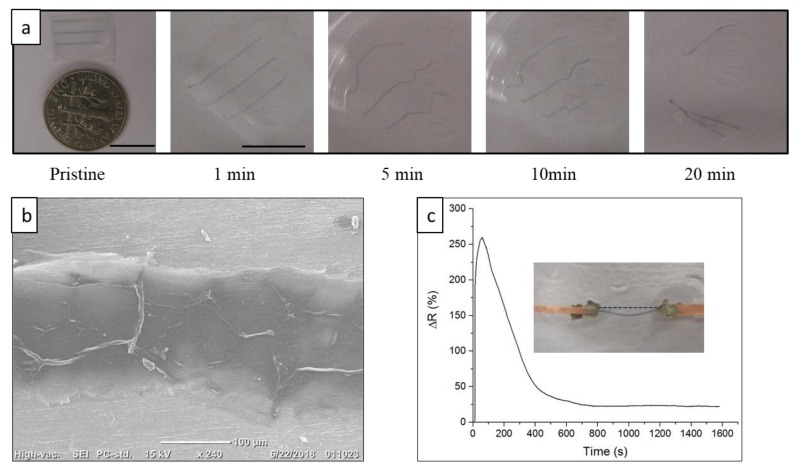
(**a**) Sequential images of transiency of all-polymer electrode in water; scale bars represent 10 mm. (**b**) SEM image of electrode with dissolved substrate, then transferred onto aluminum foil. (**c**) Electrode resistance as function of time in water; inset image is the electrode on a glass slide after all the substrates were completely dissolved.

While the PEO substrate is water-soluble, printed PEDOT:PSS electrodes are not. We demonstrated in previous studies that insoluble active electrodes might undergo disintegration by swelling-induced stress of the substrate [10,55,56,57,58]. Here, the transiency of the all-organic electrodes in water was studied and is presented in Figure 4a. As shown in the image series, the PEO substrate swelled and dissolved in water for less than 20 min, while the PEDOT:PSS electrode did not dissolve or disintegrate. The swelling-induced stress at the interface of the PEO and the PEDOT:PSS layers was not high enough to fracture the PEDOT:PSS substrate, most probably because the modulus of the swelling PEO substrate was lower than that of PEDOT:PSS. As found in our previous analysis of interfacial stress between dissimilar materials, interfacial swelling-induced stress was highly related to the ratio of elastic modulus between swelling substrate and printed layer [55,56]. The substrate-less PEDOT:PSS electrode was then transferred onto a secondary platform for SEM imaging. As presented in Figure 4b, no microcracks were observed in the PEDOT:PSS electrode.

To further investigate the electric properties of the PEDOT:PSS electrode (post-substrate transiency), resistance fluctuations of the electrode in water were monitored and recorded as a function of time. Presented in Figure 4c, normalized resistance instantaneously increased to approximately 200% as the electrode was exposed to water. Peak resistance occurred after approximately 100 s of exposure, then dropped and stabilized around 25% after approximately 700 s. The instantaneous increase in resistance was attributed to the fast swelling of the PEO substrate in water, which resulted in swelling stress, and therefore strain, on the PEDOT:PSS electrode. As the PEO substrate swelled further, relative resistance gradually increased. Subsequently, the PEO substrate started to dissolve away, and swelling stress disappeared. Consequently, the PEDOT:PSS electrode started to relax and recover the lost conductivity until it stabilized at 25%. The dissolution of the extra insulating PSS chain could be another contributor to conductivity recovery, as washing away the extra PSS chain to improve conductivity was a method used in some studies [35,59]. Conductivity was not full because the electrode did not return to its original length, as shown in the inset. Figure 4c shows that the substrate-less PEDOT:PSS electrode still retained most of its electrical properties after the PEO substrate was completely dissolved in water. On the basis of this observation, we examined substrate-less PEDOT:PSS electrodes as epidermal strain sensors after the substrates were fully dissolved.

### 3.4. Epidermal Strain Sensor

**Figure 5:**
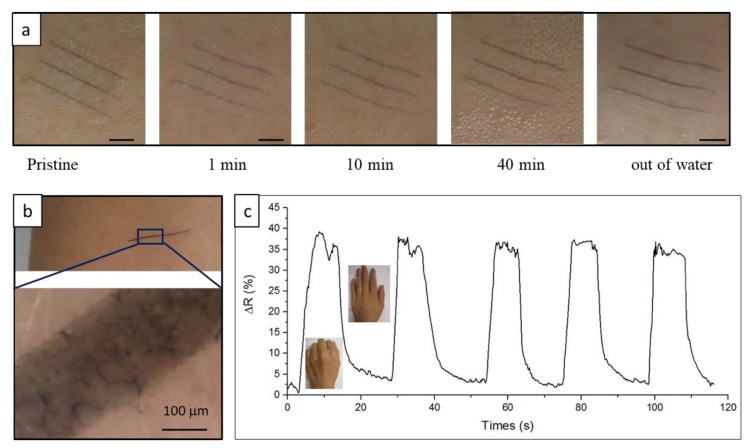
(**a**) Sequential images of transiency of all-polymer electrode on human forearm skin; scale bars present 10 mm. (**b**) Image of substrate-less electrode (after substrate was completely dissolved) on forearm skin taken with CCD camera and digital microscope. (**c**) Resistance fluctuation of pure electrode mounted on opisthenar to monitor hand movement.

In the transiency study, we found that the substrate-less PEDOT:PSS electrode, with the PEO substrate completely dissolved, still retained most of its electrical properties. The substrate-less electrode thus has great potential as an ultrathin epidermal sensor that forms perfect contact with the curvilinear surface of the host tissue (skin in this study). Researchers have reported that ultrathin graphene-based sensors that are attached to the skin with Van der Waals force could exhibit higher sensitivity than that of a thicker sensor [30]. Considering PEDOT:PSS is noncytotoxic [42,43], its application as an epidermal sensor should not pose any risk to the host tissue. To apply the sensor on skin, it was attached to a forearm; then, the PEO substrate was dissolved using water (Figure 5a). Figure 5b shows digital and microscopy images of an electrode on the skin. Conformal contact between electrode and skin are evident from the magnified microscopy image. With the substrate-less PEDOT:PSS electrode on the opisthenar, the response of the electrode to cyclic hand movement is shown in Figure 5c. The substrate-less electrode did not detach, delaminate, chip, or crack while on the skin during the examination, and it could remain on the skin for several hours. Van der Waals force was speculated to be responsible for electrode–skin bonding. A similar bonding mechanism was reported for a substrate-free graphene tattoo [30]. Studies showed that PEDOT:PSS could stably function on human-skin glucose monitoring [60] and electromyography [32], demonstrating the stable chemical and electronic properties of PEDOT:PSS on human skin. While there is a self-attached PEDOT:PSS tattoo sensor, water in excessive sweat might turn the PEDOT:PSS into hydrogel [61]. How hydration affects the contact strength between human skin and PEDOT:PSS electrode needs further study.

## 4. Conclusions

A partially transient all-organic sensor with functional PEDOT:PSS as the active electrode and a transient PEO membrane as the substrate was fabricated with an E-jet printer. To evaluate the all-organic electrode’s potential application as a flexible sensor, the electrode’s electronic properties dependence on static load and dynamic load were studied. Under a static load, resistance gradually increased within 6.5% strain. With a dynamic load, correlation between frequency and resistance was not found for frequencies ranging from 0.05 to 3 Hz, revealing a good response time of the all-organic electrodes. Correlation existed between strain and resistance, and the threshold for the reversible stretching strain was 3%. The static- and dynamic-load study demonstrated that this all-organic sensor could be applied as a flexible sensor as a whole. Meanwhile, the transiency study showed that, once transiency is triggered, the PEO substrate completely dissolved in water, while the PEDOT:PSS electrode remained almost intact. The substrate-less PEDOT:PSS electrode could form perfect contact with the skin, and was thus applied as an epidermal sensor to monitor hand motion. The partially transient property of the all-organic sensor suggests a new method to fabricated substrate-free tattoo epidermal sensors.

## Supplementary Materials

The following are available online at https://www.mdpi.com/1996-1944/13/5/1112/s1, Figure S1: Contact angle between PEODOT:PSS solution droplet on PEO substrate.
