# Supplementary material for: Study of Partially Transient Organic Epidermal Sensors
Source: Materials (Basel). 2020 Mar 2;13(5):1112. doi: 10.3390/ma13051112 (PMC7085048; doi:10.3390/ma13051112)
Supplement: Supplementary file 1 [file materials-13-01112-s001.pdf]

materials

Article

Study of Partially Transient Organic Epidermal Sensors

Supporting Materials

To evaluate the printing ability of PEO substrate, contact angle measurement was done by dropping 5 μL PEODOT:PSS solution on PEO substrate, then measured the contact angle. As seen from Figure 1, the contact angle is 55.653 °C.

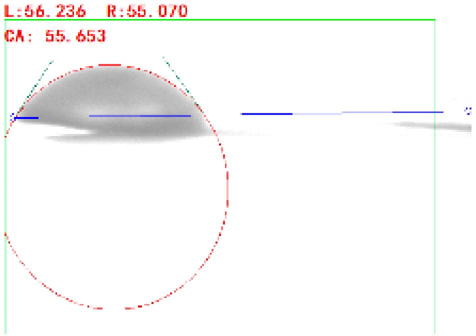

Figure S1. Contact angle between PEODOT:PSS solution droplet on PEO substrate.
